# Robotic-Assisted Resection of Transverse Colonic Leiomyosarcoma With Suspected Lymph Node Metastasis

**DOI:** 10.7759/cureus.102340

**Published:** 2026-01-26

**Authors:** Ryota Suda, Tetsuo Ishizaki, Kei Yokozuka, Munehide Nakatsugawa, Shigeyuki Kawachi

**Affiliations:** 1 Department of Digestive and Transplantation Surgery, Tokyo Medical University Hachioji Medical Center, Tokyo, JPN; 2 Department of Gastrointestinal and Pediatric Surgery, Tokyo Medical University Hospital, Tokyo, JPN; 3 Department of Diagnostic Pathology, Tokyo Medical University Hachioji Medical Center, Tokyo, JPN

**Keywords:** colectomy, colonic leiomyosarcoma, lymphadectomy, robotic-assisted surgery, transverse colon

## Abstract

Colonic leiomyosarcoma is an exceptionally rare gastrointestinal mesenchymal tumor, often misclassified before the advent of immunohistochemical markers distinguishing it from gastrointestinal stromal tumors (GIST). We report the first case of robotic-assisted surgery for a transverse colonic leiomyosarcoma with suspected lymph node metastasis. A 73-year-old male presented with abdominal discomfort. Imaging revealed intussusception due to a 3-cm tumor in the transverse colon and an 8-mm enlarged lymph node. Biopsy showed spindle cell proliferation, immunohistochemically positive for α-SMA and desmin, but negative for GIST markers. Based on these findings, a diagnosis of leiomyosarcoma (cT1N1M0, Stage III) was made. Robotic transverse colectomy with regional lymphadenectomy was performed, achieving complete resection. Pathology confirmed a 3-cm leiomyosarcoma without nodal metastasis. The patient recovered uneventfully and remained disease-free at nine months.

Although lymph node metastasis in colonic leiomyosarcoma is uncommon, effective systemic chemotherapy has not been established, and reliable preoperative assessment of nodal involvement remains challenging. Therefore, complete surgical resection remains the only potentially curative treatment, and robotic-assisted surgery may offer significant advantages by facilitating precise and meticulous operative management. To the best of our knowledge, this is the first reported case of robotic-assisted surgery for colonic leiomyosarcoma.

## Introduction

Since the establishment of diagnostic methods for detecting c-kit gene mutations, the majority of tumors previously diagnosed as gastrointestinal leiomyosarcomas have been reclassified as gastrointestinal stromal tumors (GIST). True leiomyosarcomas account for only approximately 0.1 to 10% of gastrointestinal mesenchymal tumors [1]. While the incidence of lymph node metastasis in colonic leiomyosarcoma is generally low [2], a small number of cases with nodal involvement have been reported [3]. There is currently no established method for diagnosing lymph node metastasis of colonic leiomyosarcoma using preoperative imaging. In recent years, minimally invasive approaches to colon resection have evolved from conventional laparoscopic techniques to robotic surgery [4,5]. We report a case in which robotic surgery was performed for a transverse colonic leiomyosarcoma with suspected lymph node metastasis.

This case is presented to highlight the feasibility and technical considerations of robotic resection for colonic leiomyosarcoma. To our knowledge, it represents one of the earliest reported cases of robotic surgery for a transverse colonic leiomyosarcoma, providing new insights into the surgical approach and perioperative management for this rare tumor.

## Case presentation

**Figure 1:**
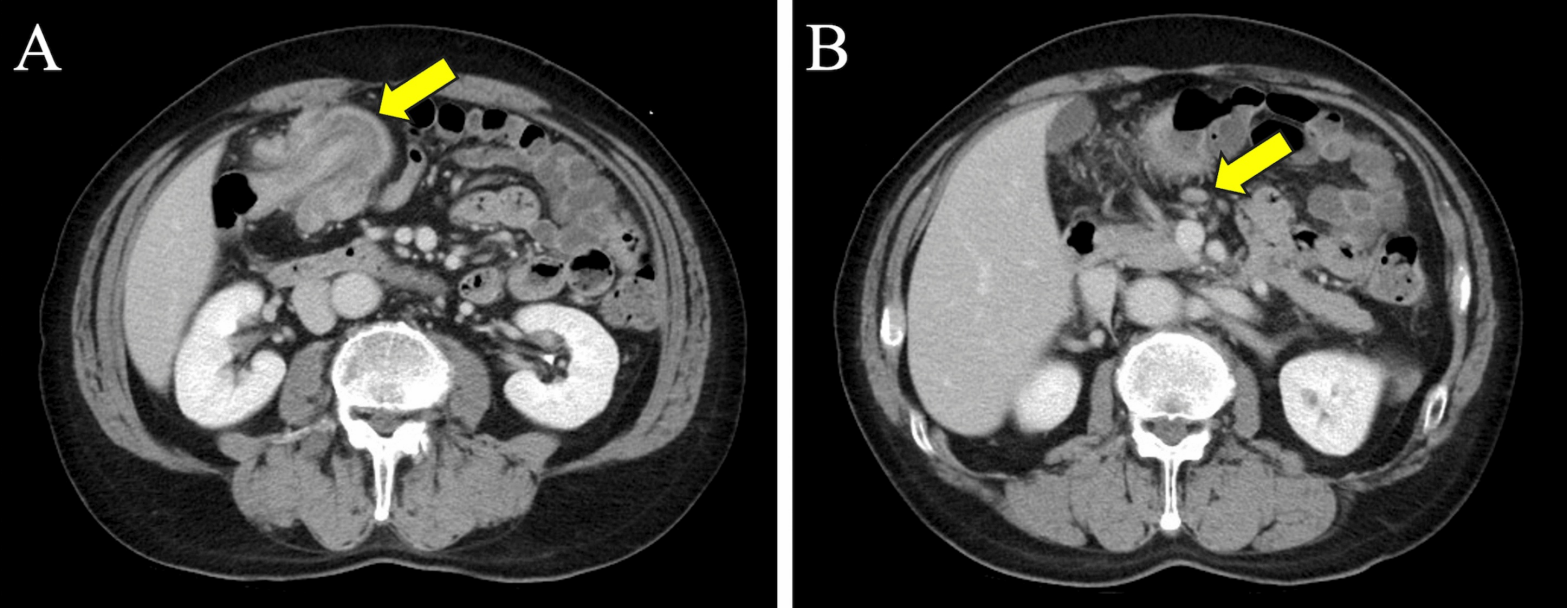
Preoperative imaging findings of the transverse colonic leiomyosarcoma (A) An intussusception with a 3-cm tumor serving as the lead point in the transverse colon. (B) An enlarged lymph node measuring 8 mm was observed around the middle colic artery

**Figure 2:**
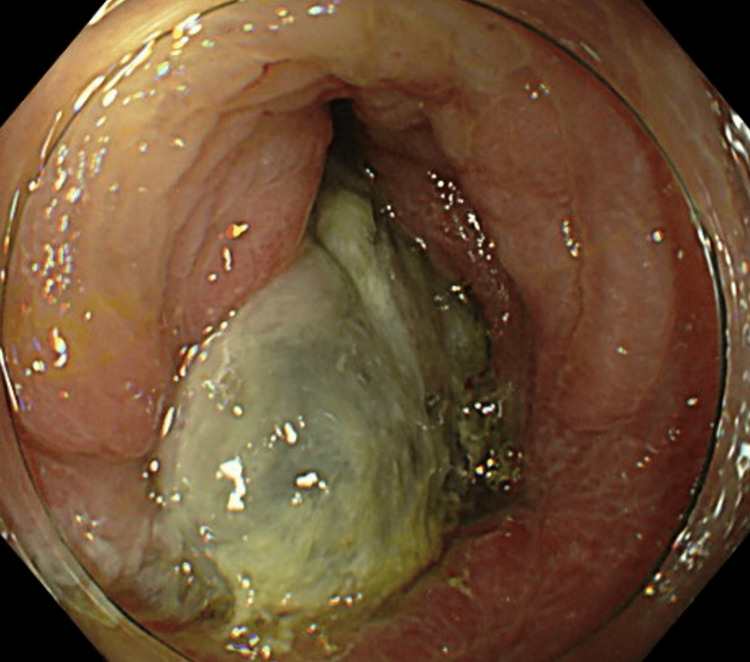
Preoperative colonoscopy findings of the transverse colonic leiomyosarcoma An irregular elevated lesion in the transverse colon along with intussusception with the lesion serving as the lead point

The patient was a 73-year-old male who presented in March 2025 to a clinic in Tokyo, Japan, with a chief complaint of abdominal discomfort. His medical history included post-appendectomy, and his American Society of Anesthesiologists physical status was class I. CT revealed an intussusception with a 3-cm tumor serving as the lead point in the transverse colon (Figure 1A), and an 8-mm lymph node was assessed by the radiologist as having a potential for metastasis (Figure 1B). Colonoscopy revealed an irregular elevated lesion in the transverse colon along with intussusception, with the lesion serving as the lead point (Figure 2). Endoscopic reduction of the intussusception was successfully performed during the procedure. Hematoxylin-eosin staining of the biopsy specimen revealed proliferation of atypical spindle cells in the submucosa. Occasional mitotic figures and areas of necrosis were also noted. Immunohistochemically, the tumor cells were positive for α-SMA, desmin (smooth muscle markers), and negative for DOG-1, c-kit, and CD34 (GIST markers), as well as S-100 (neural marker). The Ki-67 labeling index was elevated at 40%. Blood chemistry examination showed no abnormal findings, including levels of the tumor markers cancer embryonic antigen and carbohydrate antigen, which were within the normal range. According to the 8th edition of the American Joint Committee on Cancer (AJCC) 8th edition (2017) guidelines for soft tissue sarcoma, the tumor was staged as T1N1M0 (Stage III).

**Figure 3:**
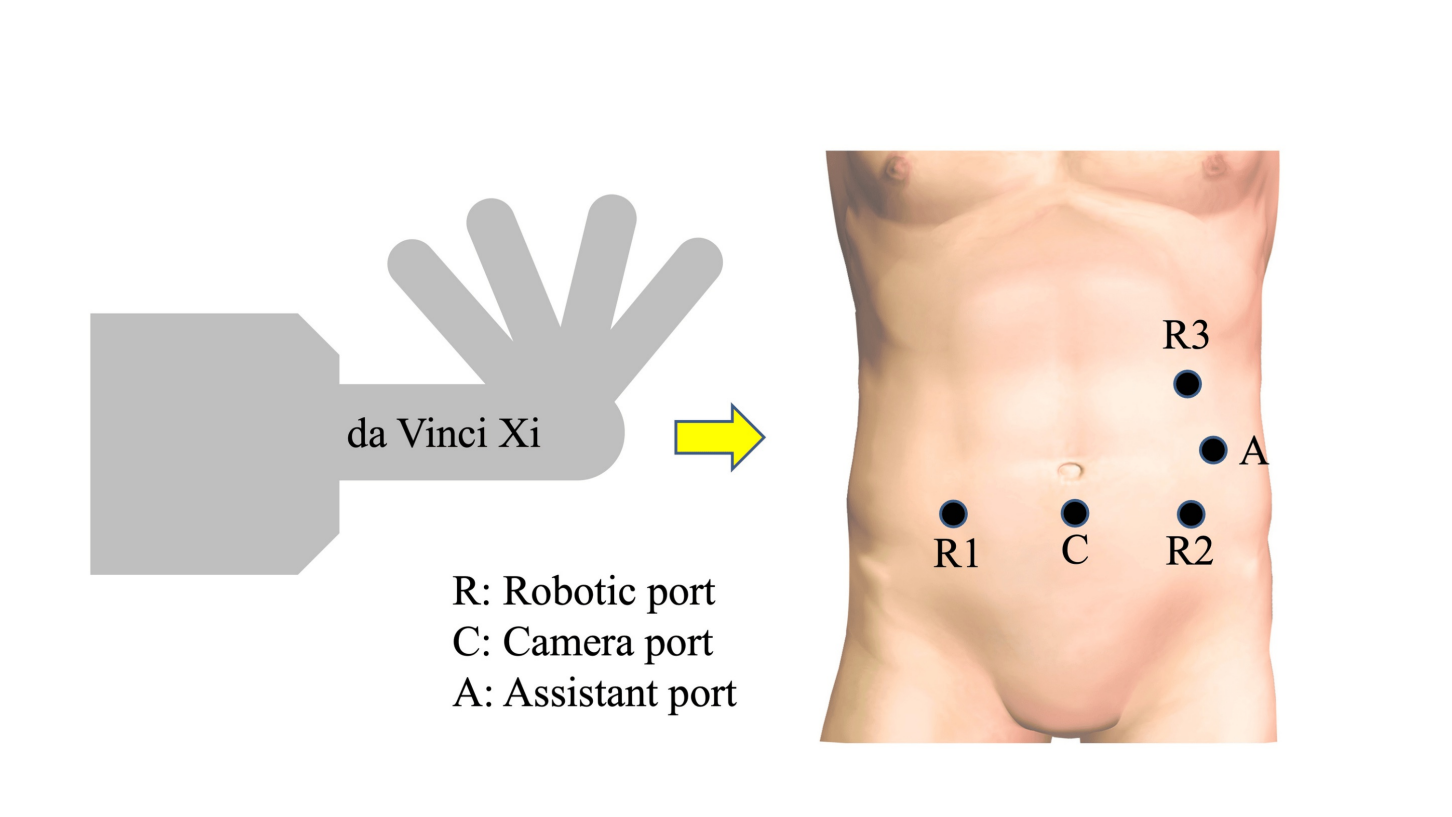
Port placement diagram The da Vinci Xi patient cart was positioned on the patient’s right side

**Figure 4:**
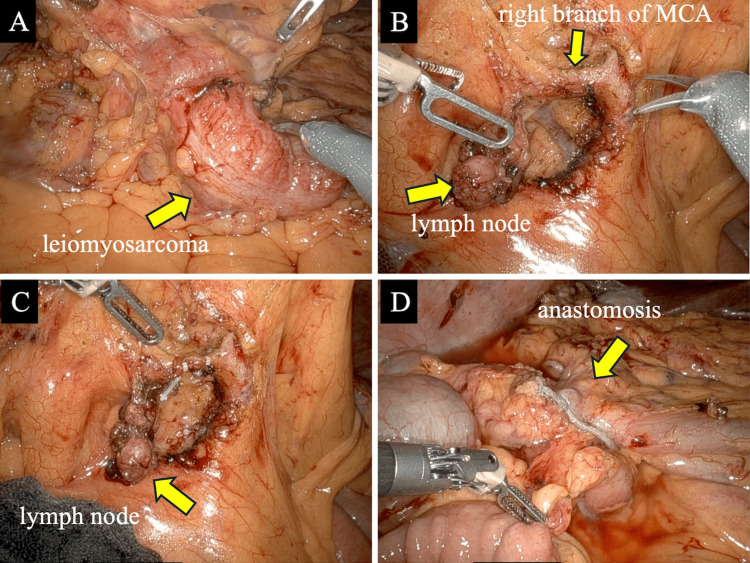
Intraoperative findings (A) A tumor was located in the proximal transverse colon. (B) An enlarged lymph node was observed at the right branch arising from the middle colic artery. (C) Lymph nodes surrounding the middle colic artery were included on the resection side, and the right branch was ligated and resected en bloc with the enlarged lymph nodes. (D) Intracorporeal overlap anastomosis was created

The decision to proceed with robotic surgery was made during a multidisciplinary conference involving our department and the radiologist, because complete resection was deemed feasible based on preoperative imaging studies. The surgery was initiated using the da Vinci Xi system, with the patient cart positioned on the patient’s right side, and a five-port approach (Figure 3). Upon exploration of the abdominal cavity, no non-curative factors were identified. A tumor was located in the proximal transverse colon. Lymphadenectomy was performed as an initial step. The transverse mesocolon was mobilized to expose and identify the superior mesenteric vein. An enlarged lymph node was observed at the right branch arising from the middle colic artery. Lymph nodes surrounding the middle colic artery were included on the resection side, and the right branch was ligated and resected en bloc with the enlarged lymph nodes (Figure 4). A 19-cm segmental resection of the colon, centered on the tumor, was performed. Intracorporeal overlap anastomosis was created, and Indocyanine Green (ICG) fluorescence imaging was used to confirm the perfusion of the anastomosis. The surgical specimen was extracted via a Pfannenstiel incision. Total operative time was 254 minutes, which included 196 minutes of console time and 12 minutes of docking time. Estimated blood loss was 5 ml.

**Figure 5:**
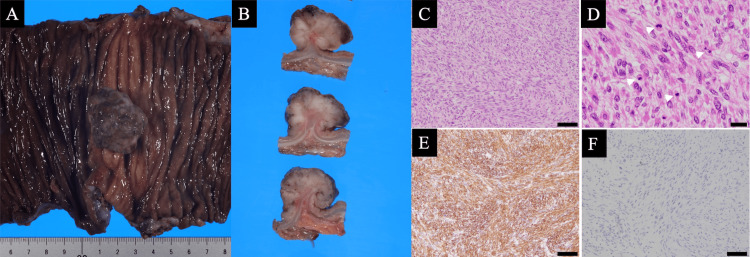
Macroscopic, histopathological and immunohistochemical findings of the tumor (A) Macroscopic examination of the resected colon specimen revealed a well-circumscribed, polypoid mass protruding into the lumen, measuring approximately 3 cm at its greatest dimension. (B) The cut surface shows a solid, grayish-white mass with a whorled appearance, arising from the colonic wall. (C) Histologically, hematoxylin and Eosin staining demonstrated that the tumor was composed of densely packed spindle cells arranged in intersecting fascicles with a characteristic whorled pattern. (D) On high-power magnification, the tumor cells exhibited abundant eosinophilic cytoplasm, blunt-ended "cigar-shaped" nuclei, and marked nuclear atypia. Numerous mitotic figures were observed, with a representative mitosis indicated by an arrow. (E) Immunohistochemically, the tumor cells showed diffuse and strong cytoplasmic positivity for α-SMA. (F) While staining for c-kit (CD117) was negative, supporting the exclusion of GIST as a differential diagnosis GIST: gastrointestinal stromal tumor

Macroscopic examination of the resected colon specimen revealed a well-circumscribed, polypoid mass protruding into the lumen, measuring approximately 3 cm at its greatest dimension (Figure 5A), and the cut surface showed a solid, grayish-white mass with a whorled appearance, arising from the colonic wall (Figure 5B). Histologically, hematoxylin and eosin staining demonstrated that the tumor was composed of densely packed spindle cells arranged in intersecting fascicles with a characteristic whorled pattern (Figure 5C). On high-power magnification, the tumor cells exhibited abundant eosinophilic cytoplasm, blunt-ended "cigar-shaped" nuclei, and marked nuclear atypia. Numerous mitotic figures were observed, with a representative mitosis indicated by an arrow (Figure 5D). Immunohistochemically, the tumor cells showed diffuse and strong cytoplasmic positivity for α-SMA (Figure 5E), while staining for c-kit (CD117) was negative, supporting the exclusion of GIST as a differential diagnosis (Figure 5F). The patient recovered uneventfully, and oral intake was started on postoperative day three. The patient was discharged on postoperative day 11. He has been disease-free and alive without recurrence for nine months postoperatively.

## Discussion

We presented a case of transverse colonic leiomyosarcoma with suspected lymph node metastasis, managed with robotic surgery that included lymphadenectomy. While robotic resection of leiomyosarcoma has been described in other organs [6], this represents, to our knowledge, the first reported case involving the colon. Gastrointestinal leiomyosarcomas most commonly arise in the stomach, followed by the small intestine, rectum, and colon. The incidence of colonic leiomyosarcoma is approximately half that of leiomyosarcoma of the rectum [7]. Clinically, leiomyosarcoma presents a significant diagnostic challenge. In addition to being far less common than colorectal adenocarcinoma, initial biopsy findings are frequently negative or inconclusive [8]. Immunohistochemical analysis is highly useful for the identification of gastrointestinal mesenchymal tumors. As α-SMA and desmin are typically positive in most cases of leiomyosarcoma, it is essential to exclude GIST and neurogenic tumors based on negative staining for CD34 and S-100 protein, respectively [9].

In this case, immunohistochemical analysis demonstrated positivity for smooth muscle markers and negativity for GIST markers and neural markers, which enabled a definitive preoperative diagnosis of leiomyosarcoma. Due to its rarity, there is no established disease classification specific to colonic leiomyosarcoma. However, staging is based on the AJCC guidelines for soft tissue sarcoma, which incorporate tumor size (T), regional lymph node involvement (N), and distant metastasis (M). In this case, the tumor was classified as c T1N1M0, corresponding to clinical Stage III. The incidence of lymph node metastasis in colonic leiomyosarcoma is extremely low [2]. However, there have been a limited number of reported cases with confirmed nodal involvement. Tago et al. [3] described a case involving a 55 mm tumor located in the transverse colon, classified as T3N1M0. Preoperative CT imaging revealed a 10 mm enlarged lymph node. Histopathological analysis showed metastasis in one lymph node dissected from a total of 14 resected nodes, a mitotic rate of 30-40 per high-power field, and a Ki-67 index of 70-80%. The patient achieved recurrence-free survival of 18 months postoperatively.

According to the most recent large-scale database study, the rate of lymph node metastasis in gastrointestinal leiomyosarcoma was reported to be 7.9% [10]. In our case, preoperative CT imaging revealed an 8-mm enlarged lymph node, comparable in size to those reported in previous studies. Currently, there is no established method for the preoperative diagnosis of lymph node metastasis in leiomyosarcoma. In contrast to GIST, effective chemotherapeutic options are lacking for leiomyosarcoma, making surgery the only potentially curative modality. Based on these findings, we determined that robotic surgery, which enables precise tumor resection and meticulous lymphadenectomy, was appropriate to enhance curative potential. 

Although minimally invasive surgery has become widely adopted in colonic procedures, previous randomized controlled trials comparing open and laparoscopic approaches excluded the transverse colon from the list of eligible surgical sites [4]. This was primarily due to anatomical complexity, the proximity of vital organs such as the pancreas and duodenum, and the technical difficulty in reflecting the hepatic and splenic flexures [11,12]. However, in the current shift from laparoscopic to robotic surgery, recent studies have demonstrated that robot-assisted transverse colectomy offers certain advantages over laparoscopic procedures, including shorter operative time, a higher number of harvested lymph nodes, and comparable complication rates [5]. Minimally invasive surgery for colonic leiomyosarcoma has rarely been reported, and all cases except the present one were managed laparoscopically. All previously reported cases were managed laparoscopically.

Previous reports indicate that patients are relatively young, that transverse colon involvement has been reported exclusively in our case, and that postoperative outcomes have generally been favorable (Table 1) [13-17]. The procedure requires a high level of surgical skill, especially in preserving the left branch of the middle colic artery while resecting the right branch in transverse resection. Robotic surgery enables precise lymphadenectomy during transverse colectomy thanks to enhanced dexterity from articulated instruments, tremor filtration, and a 3D camera system [18]. Moreover, robot-assisted surgery facilitates intracorporeal anastomosis, eliminating the need for exteriorization of the bowel and difficult mobilization of the splenic and hepatic flexures. By leveraging these advantages of robotic surgery, we believe that an optimal surgical outcome was achieved in this case. Although pathological examination revealed no lymph node metastasis, the potential for postoperative nodal recurrence necessitates appropriate lymphadenectomy in cases where metastasis is suspected based on preoperative imaging [19].

**Table 1:** Reported cases of minimally invasive surgery for colonic leiomyosarcoma

No.	Authors	Approach	Year	Age (years)	Sex	Location	Tumor size (mm)	Outcome
1	Yahagi et al. [13]	Laparoscopic	2019	46	M	Sigmoid	28	12 months, recurrence-free survival
2	Bananzadeh et al. [14]	Laparoscopic	2021	48	M	Sigmoid	80	Not described, recurrence-free survival
3	Bananzadeh et al. [14]	Laparoscopic	2021	49	M	Descending	40	Not described, recurrence-free survival
4	Wong et al. [15]	Laparoscopic	2021	59	M	Ascending	20	6 months, recurrence-free survival
5	Pagliai et al. [16]	Laparoscopic	2022	51	M	Sigmoid	60	12 months, recurrence-free survival
6	Dejima et al. [17]	Laparoscopic	2025	46	M	Sigmoid	42	42 months, recurrence-free survival
7	Our case	Robotic	2025	73	M	Transverse	30	9 months, recurrence-free survival

Leiomyosarcoma generally has a poor prognosis. According to a report by Yamamoto et al. [1], the five-year survival rate among 29 patients with gastrointestinal leiomyosarcoma was 51.6%. Tumor size more than 5 cm was identified as a significant negative prognostic factor, whereas the primary tumor site and mitotic activity did not appear to influence prognosis. Furthermore, in a study by Miyajima et al. [20] involving 115 patients with leiomyosarcoma (regardless of primary tumor location), the five-year survival rates based on the AJCC were 78% for Stage I, 49% for Stage II, 35% for Stage III, and 0% for Stage IV. In high-risk groups, neoadjuvant chemotherapy and postoperative radiation therapy may be considered to improve the rate of complete resection or to enable conversion to resectable disease [2]. On the other hand, chemotherapy may be administered for unresectable cases; however, its therapeutic efficacy remains limited [20]. Complete surgical resection is currently the only curative option for colonic leiomyosarcoma. In our case, a high-precision complete resection was achieved through robotic surgery; however, given the short observation period and the lack of established guidelines for this rare tumor, careful and continuous follow-up with CT scans every three months is required.

## Conclusions

Although lymph node metastasis in colonic leiomyosarcoma is uncommon, effective systemic chemotherapy has not yet been established, and reliable preoperative assessment of nodal involvement continues to be challenging. Therefore, complete surgical resection remains the only potentially curative option. In this report, a 73-year-old man with transverse colonic leiomyosarcoma and suspected lymph node metastasis underwent successful robotic-assisted surgical resection. Robotic surgery may offer significant advantages by enabling precise and meticulous surgical management, particularly in rare tumors such as this.

## References

[1] Yamamoto H, Handa M, Tobo T (2013). Clinicopathological features of primary leiomyosarcoma of the gastrointestinal tract following recognition of gastrointestinal stromal tumours. Histopathology.

[2] Casali PG, Abecassis N, Aro HT (2018). Soft tissue and visceral sarcomas: ESMO-EURACAN Clinical Practice Guidelines for diagnosis, treatment and follow-up. Ann Oncol.

[3] Tago T, Suzuki S, Kuroda J (2020). Leiomyosarcoma of the transverse colon with lymph node metastasis and malignant transformation: a case report. Surg Case Rep.

[4] Veldkamp R, Kuhry E, Hop WC (2005). Laparoscopic surgery versus open surgery for colon cancer: short-term outcomes of a randomised trial. Lancet Oncol.

[5] Maertens V, Stefan S, Rutgers M, Siddiqi N, Khan JS (2022). Oncological outcomes of open, laparoscopic and robotic colectomy in patients with transverse colon cancer. Tech Coloproctol.

[6] Cheng G, Ruan H, Yang C (2021). Robot-assisted laparoscopic retroperitoneal leiomyosarcoma resection with inferior vena cava graft replacement: a case report. Transl Androl Urol.

[7] Faraj W, El-Kehdy J, Nounou GE (2015). Liver resection for metastatic colorectal leiomyosarcoma: a single center experience. J Gastrointest Oncol.

[8] Sahli N, Khmou M, Khalil J (2016). Unusual evolution of leiomyosarcoma of the rectum: a case report and review of the literature. J Med Case Rep.

[9] Miettinen M, Fetsch JF (2006). Evaluation of biological potential of smooth muscle tumours. Histopathology.

[10] Bao Y, Yang X, Zhao Q, Li W (2025). Analysis of demographics and treatment outcomes for gastrointestinal leiomyosarcoma based on the SEER database. Sci Rep.

[11] Zhao L, Wang Y, Liu H (2014). Long-term outcomes of laparoscopic surgery for advanced transverse colon cancer. J Gastrointest Surg.

[12] Hirasaki Y, Fukunaga M, Sugano M, Nagakari K, Yoshikawa S, Ouchi M (2014). Short- and long-term results of laparoscopic surgery for transverse colon cancer. Surg Today.

[13] Yahagi M, Ishii Y, Hara A, Watanabe M (2019). Laparoscopic surgery to treat leiomyosarcomas of the sigmoid colon:a case report and literature review. Surg Case Rep.

[14] Bananzadeh A, Mokhtari M, Sohooli M, Shekouhi R (2021). Two cases of primary leiomyosarcoma of sigmoid colon treated with laparoscopic surgery: a case report and a review of literature. Int J Surg Case Rep.

[15] Wong GS, Yudina SV, Reyes MC (2021). Laparoscopic right hemicolectomy to curatively treat primary leiomyosarcoma at the ileocecal valve. ACG Case Rep J.

[16] Lorenzo Pagliai, Annicchiarico A, Morini A, Montali F, Virgilio E, Costi R (2022). The double challenge (preoperative diagnosis and surgical approach) of primary leiomyosarcoma of the sigmoid colon. Acta Biomed.

[17] Dejima A, Momiyama M, Nakajima K (2025). Recent treatment options for primary colonic leiomyosarcoma: a case report and a review of the literature. Surg Case Rep.

[18] Ishizaki T, Mazaki J, Kasahara K, Udo R, Tago T, Nagakawa Y (2023). Robotic right hemicolectomy with precision D3 lymph node dissection for advanced proximal transverse colon cancer-a video vignette. Colorectal Dis.

[19] Beauchamp A, Hajjar R, Khullar S, Latour M, Schwenter F, Sebajang H (2020). Mesenteric lymph node recurrence of a primary colorectal leiomyosarcoma. Case Rep Surg.

[20] Maki RG, Wathen JK, Patel SR (2007). Randomized phase II study of gemcitabine and docetaxel compared with gemcitabine alone in patients with metastatic soft tissue sarcomas. J Clin Oncol.

